# Risk factors for post-kala-azar dermal leishmaniasis (PKDL): Challenges in understanding pathophysiology

**DOI:** 10.1371/journal.pntd.0013952

**Published:** 2026-02-23

**Authors:** Eduard E. Zijlstra

**Affiliations:** Rotterdam Center for Tropical Medicine, Rotterdam, Netherlands; FIOCRUZ Bahia: Instituto Goncalo Moniz, BRAZIL

## Abstract

**Background:**

Post-kala-azar dermal leishmaniasis (PKDL) occurs mainly in Eastern Africa (EA) and the Southeast Asia region (SEAR), but with important differences. In EA PKDL occurs in young children shortly after treatment of VL (within 1–13 months) when the immune response to leishmania is developing. In contrast, in the SEAR VL and PKDL occur in adolescents and young adults with an interval of 1–4 years or more after successful VL treatment with possible downgrading of the immune response. The risk factors for PKDL in both regions are poorly understood.

**Methodology/principal findings:**

From a literature search, data was extracted with regard to risk factors, epidemiology, genetics, pathophysiology, immune responses, or co-infections in PKDL.

Among host factors, young age and male gender are risk factors in EA, and in both EA and the SEAR decreased expression of the *IFNGR1* was found in PKDL but not VL. Parasite-related factors included differences in strains of VL and PKDL as well as co-infection with symbionts such as *Leptomonas seymouri*. Previous treatment of VL was a major risk factor in Sudan, India, and Bangladesh. PK and PD of drugs used in VL and PKDL may differ. Environmental factors include naturally occurring arsenic and exposure to UV light. Lastly, co-infection with other microbes is not uncommon and may result in downgrading of the immune response after successful VL treatment in the SEAR.

**Conclusion/significance:**

A better understanding of the pathophysiology in PKDL is needed to describe factors that influence (excessive) upgrading or downgrading of the immune response. For control efforts, optimalization of VL treatment (multidrug approach, immune modifiers) may result a stronger immune response and therefore lower PKDL rates. Lastly, the approach to PKDL should be integrated in a joint skin disease approach to look for risk factors such as co-infection with a strategy for combined management and control.

## Introduction

Post-kala-azar dermal Leishmaniasis (PKDL) is a unique condition in tropical medicine; it is an intermediate disease state between visceral leishmaniasis (VL, kala-azar) and clinical cure after treatment of VL. While VL is a potentially fatal systemic disease characterized by fever, hepatosplenomegaly, pancytopenia, and weight loss in which *Leishmania* parasites may be demonstrated in all organs, in PKDL a rash develops in a patient who is otherwise healthy and has successfully recovered from VL. The clinical, parasitological, and immunological characteristics of VL, PKDL, and cure are summarized in Table A in S1 Text [1].

In addition to the clinical manifestations, PKDL has epidemiological importance because of its potential role in transmission causing outbreaks of VL (inter-epidemic) and sustaining transmission during outbreaks (intra-epidemic) [1].

In immunocompetent individuals, PKDL is restricted to patients in whom VL is caused by *Leishmania donovani* and occurs in Eastern Africa (EA), mainly Sudan and South Sudan, but also in Ethiopia, Kenya, Uganda, and Tanzania; and in the Southeast Asia Region (SEAR), mainly India, Bangladesh, and Nepal. In immunocompromised individuals, PKDL is also associated with infection by *L. infantum* [2].

Differences exist in epidemiology, clinical presentation, and management of PKDL in the SEAR and EA. (Table 1). In EA, the most striking features are the very young age at presentation with PKDL developing within 13 months of VL treatment, likely the result of a developing immune response to leishmania. In contrast, PKDL in the SEAR occurs in adolescents and young adults after a mean interval of 3 years, sometimes up to 15 years after VL treatment suggesting a re-infection or a breakdown of the immune response to Leishmania.

**Table 1:** Characteristics of PKDL in Southeast Asia Region (SEAR) and Eastern Africa (EA): factors related to epidemiology, clinical features, and management (adapted from [3].

	SEAR	EA
Frequency after VL	10% (India) 20% (Bangladesh)	Early data 50–60%; more recent 2–20% (Sudan)*
Age group (most common)	Adolescents, young adults	Young children (1–10 years)
Most important clinical presentation	Macular rash 90%	Papular rash 90%
Interval after VL (most common)	1- >4 years (peak at 3 years)	0–13 months
Self-healing	Uncommon	Common, in 85%
Current treatment policy	All are treated	Only severe and chronic cases**
Possible role in transmission	Yes	Yes

*For an overview: Table B in S1 Text.

**New guidelines expected in 2026.

### Clinical entities

Post-kala azar dermal leishmaniasis (PKDL) is the classical and most common presentation and is characterized by the occurrence of macules, papules or nodules after apparently successful treatment of VL. In Asia, the macular presentation is most common in field studies while in hospital-based studies, the polymorphic form is more common. In Africa, a papulonodular rash is most common. The face is often affected first, and the disease may spread to other parts of the body with variable distribution and severity; a clinical grading system was developed for PKDL in Sudan [4]. Images of PKDL and differential diagnoses from various geographical locations may be accessed in WHO publication “The Post-Kala-Azar Dermal Leishmaniasis (PKDL) Atlas” (WHO, 2012) [5].

**Fig 1:**
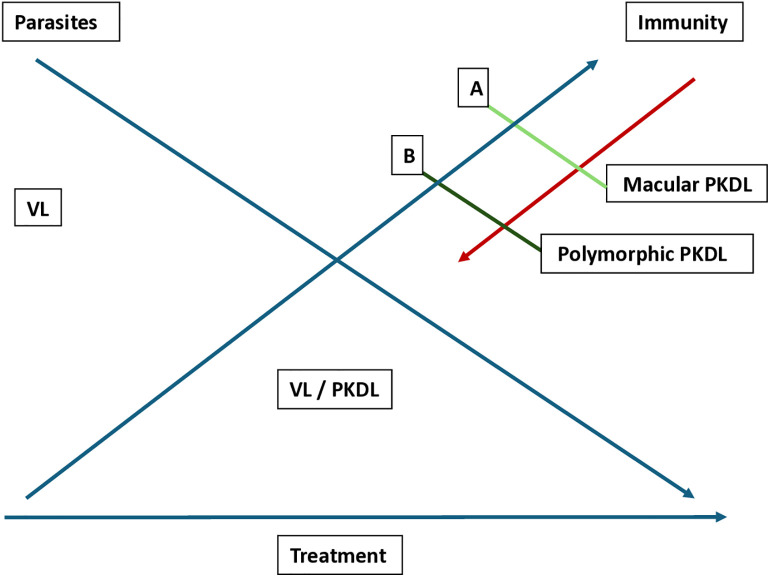
Adapted with permission from ref [81]. Hypothetical relationship between decreasing parasite load and increasing immunity. In case of downgrading of the immune response (red arrow), PKDL is likely to result in a macular rash first with stronger immune response (**A**, higher CMI*, scanty parasites) than a polymorphic or papulonodular rash (**B**, lower CMI*, higher number of parasites). In case of upgrading of the immune response the opposite would occur. * CMI cell-mediated immunity.

There are three other less common manifestations:

- Para-kala-azar dermal leishmaniasis occurs when PKDL already develops during VL treatment; in addition to the rash, the patient may have fever, hepatosplenomegaly and poor nutritional status. Parasites may be demonstrated in the lymph nodes, bone marrow or spleen as well as in the skin [1]. (Fig 1)
- PKDL without previous VL occurs in 10% of PKDL cases; this probably means that VL has not been recognized as an infection and may have been asymptomatic. Alternatively, in case of clinical features such as fever and splenomegaly these may have been misinterpreted as, e.g., a malaria episode or an alternative diagnosis according to the geographical area [6].
- PKDL-like lesions in VL-HIV co-infection may occur preceding, during or after VL infection or treatment; dermal lesions in HIV-VL co-infection may be a more appropriate term [2]. Both *L. donovani* and *L. infantum* may be causative agents [5].

The evidence regarding risk factors for developing PKDL including the variable clinical characteristics in each endemic region is inconclusive.

We examine the epidemiology, clinical features, and pathophysiology of PKDL in the SEAR and EA with the aim to identify risk factors, relevant gaps in knowledge, and research priorities.

## Methodology

We searched PubMed using the following search criteria: (post-kala-azar dermal leishmaniasis or PKDL) and (risk factors *or* epidemiology *or* genetics *or* pathophysiology *or* immune responses *or* co- infections *or* drug treatment). All papers in the English language up to the year 2025 were included. Additional papers from the author’s own collection were also included.

## Risk factors

### Host factors: Age, gender, malnutrition, genetics

#### Age and gender.

In EA, VL and PKDL occur in young children, in age group between 1 and 10 years [7,8]. In field-based studies, the male-to-female ratio is 1.8:1, while in case series the ratio is closer to 1:1, possibly explained by outdoor activities of boys including herding cattle and spending the night in the field, while girls spend most of the time around the house [8–10].

In the SEAR, VL occurs mainly in young adults with PKDL following 1–>4 years later; in one study, the age range of PKDL patients was 5–24 years [11]. While in hospital-based studies in nonendemic areas a male preponderance (80%) is reported among PKDL patients with a male-to-female ratio of 3–4:1, in endemic areas, the ratio seems much more balanced (ratio 1.2–1.9) and not different from that found in VL [12]. However, a male predominance was demonstrated in PKDL patients (in contrast to VL) that was found to be associated with high testosterone levels and high levels of antileishmanial IgG [13]. No space but continue with next sentence: Testosterone has been ...

Testosterone has been shown in animal studies to influence the immune response resulting in increased susceptibility and severity of disease, with increased uptake of parasites by macrophages. Other factors may include epigenetics and gene expression [14].

#### Malnutrition.

Recently, the importance of malnutrition was emphasized as a potential risk factor for PKDL in India and providing nutrient-rich food during VL treatment was suggested [15].

#### Genetic studies.

Host genetic variations in leishmaniasis have mainly been studied in relation to the immune system including genes of metabolic processes; these were recently reviewed [16]. While many candidate genes have been identified, genome-wide, well-powered studies are needed [16].

PKDL in Sudan was found associated with decreased function of the interferon-gamma receptor 1 gene [*IFNGR1*]) that is not found in VL [17]. PKDL skin biopsies showed uniform low expression of IFN-γ and *IFNGR1*, that may explain the persistence of parasites. In Indian PKDL, similar downregulating of the IFN-γ response at mRNA and protein level was found in PKDL biopsies despite high levels of IFN-γ and TNF-α, due to minimal expression of *IFNGR1* or the simultaneous presence of counteracting cytokines including IL-10, IL-6 and TGF-β. These abnormalities were restored after treatment [18,19]. No polymorphisms were demonstrated for the IL-10 promotor gene [19].

Transcriptomics describes the immunological response to infection by assessing the gene expression (“signature”) in the interaction between host and pathogen. In a study from India, using whole blood transcriptional profiling, the gene expression profile of active VL was demonstrated to be different compared with healthy controls. In addition, support was found for more effective cure at 30 days post-treatment for single-dose AmBisome vs. multi-dose conventional amphotericin B for 30 days [20]. The implications for the occurrence of PKDL are not clear.

### Parasite-related factors

PKDL seems to be restricted to VL caused by *L. donovani* and this determines the geographical distribution of PKDL that occurs mainly in the SEAR and EA. It is rare in *L. infantum* infection except in VL-HIV co-infection [1,2].

#### Differences in strains of VL and PKDL.

Early studies in the SEAR show no convincing evidence that strains causing VL and PKDL differ, but there are few data on paired strains and most studies are cross-sectional. In addition, only minor differences in strains from VL and PKDL have been demonstrated so far, except for drug sensitivity. However, some proteins are upregulated such as Gp63 and Promastigote Surface Antigen (PSA), that may alter the parasite and promote accumulation in the skin [21].

In a study from India that focused on the genetic variations between VL and PKDL-causing strains, nuclear DNA from 24 strains of *Leishmania donovani* was isolated from patients of visceral leishmaniasis (18 patients) and PKDL (6 patients). Among the 18 VL strains three different banding patterns were shown; the PKDL isolates showed a genetic homogeneity within themselves but heterogeneity from VL isolates. Interestingly, maximum heterogenic groups were found in Bihar, but all isolates from West Bengal showed a single genotype origin. This study shows that genetic mutations might be responsible for such variation and development of PKDL in visceral strains of Indian *L. donovani* [22]. Genetic diversity was also shown in *L. donovani* isolates from VL and CL patients in Kerala, India, that were different from isolates collected elsewhere but closely related to isolates from India and Sudan [23].

Using whole-genome sequencing, clustering of 24 unique genes was demonstrated in strains of para-kala-azar dermal leishmaniasis patients, that could possibly be linked to strains becoming dermatotropic [24,25]. In cultured dermal fibroblasts from VL and PKDL patients 516 differentially expressed genes (DEGs) were found (263 overrepresented and 253 underrepresented) in transcriptome analysis. This suggests that PKDL fibroblasts may present antigens through the MHC I pathway activating CD8+ T-cell mediated response while in VL antigen expression leads to recruitment of natural killer cells and monocytes to the site of infection, resulting in clearance of parasites from the skin and visceralization [26].

#### Symbiontic co-infection in Leishmania parasites.

Other parasite-related factors include co-infection with Leishmania RNA virus 1 (LRV1), that has been associated with treatment failure in CL caused by *L. guyanensis* and the subsequent occurrence of mucosal leishmaniasis [27]. Experimental data suggest the virus promotes inflammation and subverts the immune response leading to a Th2 predominant cytokine profile. *Leptomonas seymouri* is considered non-pathogenic to humans; however, it may exist as a co-infectant, e.g., in *Leishmania* spp. probably after adaptation from a monoxenous to dixenous life cycle. It has been found in samples of VL and PKDL, and recently in CL patients in Himachal Pradesh, India, where CL is caused by *L. donovani* [28]. While LRV has not been demonstrated in VL samples, the *Leptomonas seymouri* narna-like virus 1 (Lepsey LRV) was recently demonstrated in VL and PKDL isolates from India and has also been found in skin biopsies (PKDL) and peripheral blood (VL). Cytokine profiling showed elevated IL-18 levels that induces a Th1 response and macrophage activation [29]. A new non-LRV RNA virus leishbunyavirus was described that infects *Leishmania martiniquensis*, with yet unclear clinical implications [30].

### Previous treatment of VL, immunomodulatory drug effects, drug penetration in the skin

#### Eastern Africa.

In contrast to the SEAR, sodium stibogluconate (SSG – Pentostam) was used much longer as the only available treatment and resistance to SSG has not been an issue as response to treatment in VL remained satisfactory. Early reports (1991–1995) showed a PKDL rate of 50–60% (after 12 months follow-up) including home-based treatment with adulterated SSG. A black market for SSG was flourishing as the only drug available was very expensive, and counterfeits were sold, consisting of just water instead of SSG. Parents would buy a few milliliters of the drug - too small to be effective - and inject the drug themselves because they could not afford to pay a nurse to do the job. This practice may also have led to high infection with hepatitis B and C virus [31,32].

Later studies (1995–2020) showed PKDL rates of 3–20% - mainly in randomized controlled trials (RCTs) with strict inclusion criteria, and supervised administration; follow-up was limited to 6 months. (Table B in S1 Text)

#### SEAR.

In the SEAR, the relationship between previous treatment of VL and subsequent development of PKDL was extensively studied.

In India, a phase IV trial included 1750 patients treated for VL who were followed up for 24 months to determine the PKDL incidence. The MF + PM combination had a higher PKDL incidence compared SDA (single dose AmBisome); the risk of PKDL was higher in children <12 years and females. The median time to onset of PKDL was 25.4 months and 95% of PKDL cases occurred by 43.1 months post-treatment [33] (Table C in S1 Text).

In Bangladesh, 974 treated VL patients were followed for 4 years; 121/984 (12.3%), developed PKDL (95% CI, 10.4%–14.5%) after a median time 2.6 years (IQR, 1.84–3.12). The incidence rate was 14.0 (95% CI 8.6–22.7). The SSG cohort had the lowest incidence rate of PKDL at 3.0 (95% CI 1.3–7.3) followed by multi-dose AmBisome at 8.2 (95% CI 4.3–15.7) [34] (Table D in S1 Text).

#### Immunomodulatory drug effects.

There is increasing evidence on immunomodulatory drug effects of antileishmanial drugs [3]. In vitro the response to drugs is host cell dependent and both the innate and adaptive immune responses are influenced [35,36]. In one comparative study in patients treated for VL, AmBisome had a stronger effect than SSG in modulating a Th2 response by reducing IL-10 and TGF-β which may reduce the risk of developing PKDL [37].

#### Drug penetration in the skin during VL and PKDL.

Recent insights from the SEAR show that miltefosine has substantial penetration in the skin after oral administration resulting in substantial exposure and prolonged retention in the skin that should be adequate to influence leishmania parasites [38,39]. This would support the use of MF in treatment of PKDL. In contrast, bioavailability of MF in VL patients was lowered by 69% at treatment start and similar reduction was found for paromomycin (0.74– to 0.87-fold reduction) compared to PKDL patients who have different clinical characteristics (disease restricted to the skin, no systemic features, no weight loss—see Table 1).

### Environmental exposure

#### Ecological differences in the endemic area.

In EA, observations from field studies suggest that differences may exist between populations in the same endemic area. In Gedaref State, Sudan, most villages are inhabited by the Masaleet tribe originating from El-Geneina, in Darfur, western Sudan; incidence of VL and PKDL rates is high and consistent across these villages [7]. In contrast, the villagers of Mushrau Koka (MK) village originate from Kano or Sakatu, in northern Nigeria, and are of the Hausa tribe. The village of MK is only 30 kilometers separated from Um-Salala, but with a different ecology compared to Um-Salala: more sandy soil, predominance of the neem tree (*Azadirachta indica*) rather than *Acacia seyal* and *Balanites aegyptiaca* vegetation, and less abundance of sand flies. In a comparative study the incidence of VL is much lower than in Um-Salala (62 and 7 cases in two-years observation, for Um-Salala and MK, respectively, while the LST positivity rate as a measure of exposure to *Leishmania* was similar suggesting that in addition to difference in ecology, other factors such as ethnicity and nutritional parameters may be of importance [31].

#### Arsenic.

In the SEAR, arsenic occurs in groundwater, which is an essential source of drinking water, causing a health hazard for those exposed. Chronic arsenic toxicity (CAT) results in several genotoxic and epigenetic alterations resulting in, among others, in skin disease with keratosis that emerges as diffuse thickening of palms and soles, alone or in combination with nodules [40]. CAT was demonstrated to be a risk factor for PKDL that was more common in high baseline arsenic exposure [41].

#### UV light.

The distribution of PKDL lesions in both EA and SEAR often initially mirrors the clothing habits of those affected. It is most severe in or restricted to sun-exposed parts of the skin. The elimination of *Leishmania* parasites requires activation of parasitized macrophages by a Th1 immune response and the latter is depressed by ultraviolet light (UVB). It is probable that UVB is a a key factor in the pathogenesis of PKDL; it may select those who develop the disease as 40% of population are sensitive to UVB light. Clinically, this explains the occurrence of PKDL lesions on the face as a first presentation, although this seems less applicable to macular lesions, both in PKDL in EA and the SEAR [42].

### Re-infection with Leishmania; intercurrent or co-infections with other pathogens

#### Re-infection—Sand fly related factors.

People living in an endemic will be repeatedly exposed to sand fly bites. Co-infection in sandflies of leishmania parasites with *Leptomonas seymouri* narna-like virus (Lepsey narna) may occur as well as co-infection with other viruses such as Sandfly Fever Sicilian Virus (SFSV) and Toscana virus. In India, in addition to *Phlebotomus argentipes*, other sand flies have been incriminated in transmission of *L. donovani* such as *P. papatasi*, *P. sergenti*, *P. longiductus*, *P. bruneyi*, and *P. major*. High infection rates were shown for *P. argentipes* and *P. longiductus* [43]. For each, biological habitat, biting behavior, inoculum, co-infection, and influence of saliva needs to be determined in effectiveness of transmission and the subsequent immunological response of the patient.

In EA, *P. orientalis* is the prominent vector in Sudan and northern Ethiopia that is highly exophilic and exophagic; it is a highly seasonal species with abundance in March-June at the onset of the heavy rains and it thrives in remote woodlands characterized by black cotton soil with *Acacia seyal* and *Balanites aegyptiaca* trees. In contrast, *P. martini* is the principal vector in South Ethiopia, South Sudan, Kenya, and Uganda; its habitat are termite hills. Here, PKDL is much less common compared to Sudan. It is not certain to what extent the vector species contributes to PKDL in addition to other factors such as different population, *Leishmania* spp., or ethnicity [44].

#### Intercurrent or co-infections with other pathogens.

There are numerous reports on intercurrent or co-infections in VL or PKDL, including parasitic, viral fungal, and bacterial pathogens. While most are (cross-sectional) case reports, other reports comment on the influence of alterations in the immune response from a first microbial infection on acquiring a second infection by another microorganism including implications for clinical presentation or pathophysiology (Table 2) [45].

**Table 2:** Co-infections in VL and PKDL: groups of pathogens, interaction, presence of immunosuppression and country/region where reported.

Viruses	Interaction	Immunocompromised	Endemic region	Reference
• HBV	VL induced immunosuppression reactivates silent HBV infection; after VL treatment hepatic necrosis because of immune restoration?	No	Italy	[57]
• HBV/HCV	Among 78 VL with co-infection, 67% had HBV, 27% HCV, and 6% HBV/ HCV High AST and low albumin point to on-going liver damage	Unknown	Sudan	[58]
• HBV/ HCV	High prevalence of HBV (16%) and HCV (14%) among VL patients in India and Sudan	Unknown	Sudan, India	[59]
• HCV	Reactivation of VL after direct-acting antiviral drugs (DAAs)	No	Italy	[60]
• HIV	Mutually increase severity; PKDL-like skin lesions preceding, during, or after VL	Yes	world-wide	[2,61,62]
• Epstein Barr Virus (EBV)	Co-infection with EBV-induced secondary hemophagocytic lymphohistiocytosis	No	Spain	[54]
• Cytomegalovirus (CMV)	Co-infection with unexplained low CD4 count	No (HIV neg)	India	[63]
• Covid-19	*L. infantum* is risk factor for Covid-19	No	Iran	[64]
• Measles	Recurrence of VL after measles infection in PKDL	No	India	[65]
• *Leptomonas seymouri*	Co-infection in VL and PKDL	No	India	[66]
• *Leptomonas seymouri* narna-like virus	Co-infection in VL and PKDL; parasite burden may increase in VL	No	India	[29,67]
Bacteria				
• *B. melitiensis*	Co-infection in VL and PKDL, suggestion of the goat as common factor	No	Keyna	[68]
• *F. tularensis*	Leishmanial (presumed *L. infantum*) lymphadenopathy (biopsy) and tularemia (serology, 4-fold increase in titers)	No	Armenia	[69]
• *M. leprae*	Co-infection; the case described developed PKDL after VL and leprosy treatment	No	Brazil	[70]
• *M. leprae*	Co-infection in PKDL	No	India	[71]
• *M. tuberculosis*	Co-infection with HIV-VL leads to high mortality	Yes	Ethiopia, India	[49]
• Gut bacteria	Co-infection, dysbiosis	No	India	[72]
Fungi				
• *Trichophyton rubrum* – Tinea corporis • *Malassesia furfur -* Tinea versicolor	Tinea co-infection in PKDL may disappear with anti-leishmanial treatment only	No	India Sudan	[73]
Parasites				
• *W. bancrofti*	*W. bancrofti* infection associated with progression from asymptomatic to clinical VL	No	India	[74]
• *Malaria*	Co-infection causes more severe clinical features including emaciation, jaundice, and anemia with higher risk of adverse outcome	No	Somalia	[67,75]
• *Plasmodium falciparum (P.f.)*	Both parasites induce (different) immune responses; reduced levels of P.f. parasitemia in co-infection	No	Sudan	[76]
• *Crithidia* spp	Co-infection	Yes (HIV pos)	Brazil	[77]
• *Schistosoma mansoni*	Co-infection; differential diagnosis of massive splenomegaly	No	Brazil	[45,78]
• *L. chagasi*	Co-infection	No	Brazil	[79]
• *Strongyloides stercoralis*	Fulminating co-infection in VL	No	India	[48]
• *Taenia solium*	Co-infection on the tongue	No	India	[80]

Microorganisms influence the immune system in various ways, including immune cells such as CD4+ T-cells and macrophages [46]. Co-infection may result in induced immunosuppression, elimination of immune memory, modification of the immune response, facilitating a second co-infection, or other unidentified mechanisms.

In HIV infection immunosuppression is well described and may be profound, and HIV and VL mutually influence each other in terms of risk of acquiring and clinical course [47]. PKDL-like lesions may occur preceding VL, concomitant with VL, or following VL [2]. VL may aggravate tuberculosis with high mortality and has been described to facilitate fulminant strongyloidiasis infection [48,49].

VL and PKDL may also facilitate co-infections limited to the skin; dermatophytic infections such as *Trichophyton rubrum* have been described in PKDL and may be acquired because of depressed cellular immunity; in Sudan, infection with Tinea versicolor (caused by *Malassesia furfur*) was diagnosed during VL which disappeared after treatment with sodium stibogluconate alone suggesting VL-related immune suppression as the underlying mechanism [50].

In other co-infections, immune changes may be induced that are not well characterized and may vary from subtle, or profound; e.g., measles may cause elimination of previous immune memory, making the subject again susceptible to previously encountered infections [51]. In a case report recurrence of VL after PKDL occurred after measles infection [52].

The influence of EBV on the immune system are pleiomorphic. In addition to its oncogenic potential (e.g. Burkitt’s lymphoma in co-infection with malaria), high EBV viremia has been described as co-infection with *L. infantum* in the hemophagocytic lymphohistiocytosis syndrome (HLH); similar high viremia seems implicated in the pathogenesis of multiple sclerosis in certain HLA types [53,54]. Helminthic gastrointestinal infections induce Th2 responses and may suppress immunity to viruses, bacteria and protozoa [55,56].

## Discussion

The pathophysiology of PKDL is dominated by the immune responses and factors that influence these.

**Fig 2:**
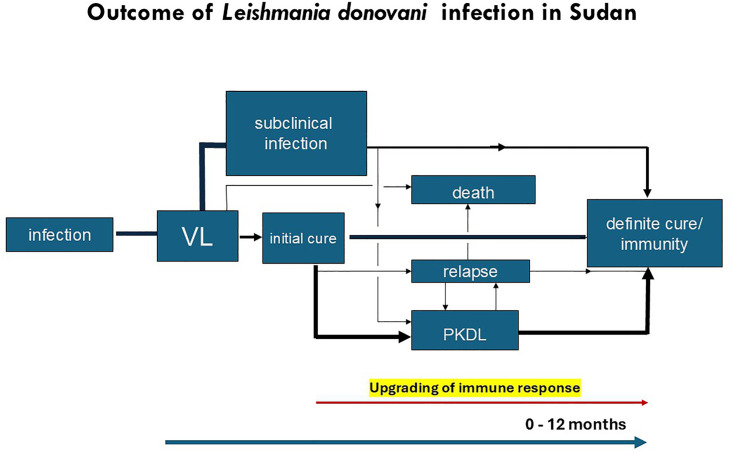
Diagram showing the interrelationship and timing of manifestations that may follow after leishmania infection in Sudan and the upgrading of the immune response preceding PKDL. The thickness of the lines corresponds with the likelihood of the occurrence of the following event (adapted with permission from reference [81].

In East Africa, the immune response is best described by gradual upgrading of an anti-leishmanial immune response after VL treatment. This is supported by most PKDL occurring within 12 months after VL treatment. In addition to young age, the VL treatment regimen has been shown to play an important role. In this context, the predominance of papulo-nodular infections may be understood as these reflect a stronger immune deficit than macular lesions. In Sudanese PKDL, the LST may be taken as a semi-quantitative marker as the test is typically negative in VL and becomes positive in 30% of PKDL cases while the positivity rate decreases with severity of PKDL. (Figs 1 and 2)

**Fig 3:**
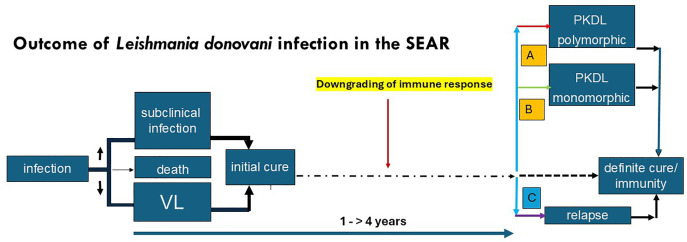
Diagram showing the interrelationship and timing of manifestations that may follow after leishmania infection in the SEAR and the downgrading of the immune response that may precede the onset of PKDL. The thickness of the lines corresponds with the likelihood of the occurrence of the following event. Box A, B, and C constitute risk factors (previous drug treatment, genetic and environmental factors, immune suppression, others) that may be qualitatively different.

In the SEAR, there seems to be a subacute downgrading of a previously successful immune response by an unknown trigger, after treatment of VL that occurs in young adults; while the VL treatment is of influence in India and Bangladesh, the (very) long interval after VL suggests that other factors may be important. The strength of the immune response after VL treatment cannot be quantified; similarly, it is not known to what extent repeated exposure to leishmania infection contributes to a sustained and protective response. The most reliable tool would be the LST as a positive LST result 12 months after VL treatment in absence of PKDL is associated with protection. Intercurrent infection that may downgrade the immune response seems likely as infections with viruses, bacteria, and parasites in low-resource settings are common. This is supported by studies that describe the changes in immune responses between VL mono-infection and co-infection; here, the predominance of macular lesions may be understood as these are likely to occur first when immunity decreases. In macular PKDL, the CMI is strong with few parasites and low antibody levels (only Ig1 is elevated) [3,82]. Further spread of initially localized macular lesions may occur and polymorphic lesions start to appear over time that become gross and extensive in HIV co-infection where immunity further decreases. In the polymorphic form (papulo-nodular) the CMI is low, induced by TGF- β and IL-10, with higher levels of markers for regulatory T cells, more parasites, and high antibody levels, including both Ig1 and Ig3 (markers for IL-10) [2,3]. It should be noted that not all co-infections have an adverse effect, but may also be protective as in co-infection with malaria. (Figs 1 and 3) (insert ref 76)

### Way forward

The PKDL dilemma persists as patients with mild lesions are reluctant to embark on a lengthy oral treatment (in the SEAR) or a combination of oral and parenteral drugs (in EA). In addition, in EA these will self-heal in 85%; in the SEAR self-healing is reported shortly after VL, but it assumed that PKDL after a long interval after VL does not self-heal [8,83]. The epidemiological impact is, however, important as untreated PKDL patients may act as a reservoir for transmission.

PKDL has been incriminated as a trigger to epidemics of VL or maintaining transmission in interepidemic outbreaks, both in EA as in the SEAR. Recent xenodiagnosis studies confirm that sand flies feed on PKDL patients, but also on patients with VL thus suggesting an alternative reservoir [84].

Currently, all PKDL patients in the SEAR are treated with miltefosine for 3 months for reasons of controlling transmission. Most are detected by active case-finding and are reluctant to start treatment for what seems only a mild rash. The risk of drop-out is considerable as miltefosine has considerable side-effects, mostly gastrointestinal (vomiting), and recently eye disease has been a reason for concern [85]. In addition, in females of the child-bearing age, reliable contraceptives are needed as the drug is teratogenic.

In EA, until recently, only severe PKDL cases were treated or those with persistent rash for > 12 months. This will be changed to treatment of all cases to interrupt transmission. Recently, a combination treatment of paromomycin and miltefosine showed good efficacy and safety [86].

In conclusion, research is needed to identify what triggers the downgrading immune response in SEAR PKDL, and what determines severity and persistence in EA PKDL. First, detailed genetic studies on PKDL strains in comparison with strains isolated from VL patients would be useful, including the role of symbiotic infection. PKDL patients should be studied as to the prevalence of intercurrent infection, underlying conditions such as malnutrition, environmental exposure, and genetic background in comparison to those who do not develop PKDL. Second, prevention of PKDL is of paramount importance. The drug treatment of VL should be optimized for an optimal and protective immune response to prevent PKDL as well as relapse of VL, with measurable biomarkers. This may include new, more effective drugs, multidrug therapy, and an immune modifier, a drug or a vaccine [87,88]. Comparative studies should include immune responses in each regimen. These studies should also include penetration in the skin and the effect on immune responses in the skin vs systemic responses.

Lastly, the Second WHO Global Meeting on Skin NTDs emphasized the integration of prevention and treatment of conditions such as PKDL with other programs that address (neglected tropical) skin diseases in terms of surveillance, clinical management, and control activities [89,90].

**Box learning points**

- Post-kala-azar dermal leishmaniasis (PKDL) is an intermediate disease entity between treatment of VL and clinical cure, restricted to *L. donovani* in immunocompetent individuals.
- PKDL (and VL) may play a role in transmission of VL.
- PKDL in Eastern Africa (EA) and the Southeast Asian Region (SEAR) differ in epidemiology, clinical features, and natural history.
- PKDL in EA may be the result of an upgrading immune response as a result of recent VL treatment; PKDL in the SEAR may be the result of downgrading of an already established immune response years after successful VL treatment.
- Factors related to age, genetics, environment, parasite, VL drug therapy, and intercurrent infection may play a role.
- Focus of research should be on better characterization of VL and PKDL patients, and not restricted to *Leishmania* infection.

**Key papers**

- Zijlstra EE, Musa AM, Khalil EA, el-Hassan IM, el-Hassan AM. Post-kala-azar dermal leishmaniasis. The Lancet Infectious diseases. 2003;3(2):87–98.
- Musa AM, Khalil EA, Raheem MA, Zijlstra EE, Ibrahim ME, Elhassan IM, et al. The natural history of Sudanese post-kala-azar dermal leishmaniasis: clinical, immunological and prognostic features. Annals of tropical medicine and parasitology. 2002;96(8):765–72.
- Islam S, Kenah E, Bhuiyan MA, Rahman KM, Goodhew B, Ghalib CM, et al. Clinical and immunological aspects of post-kala-azar dermal leishmaniasis in Bangladesh. The American journal of tropical medicine and hygiene. 2013;89(2):345–53.
- Nandy A, Addy M, Maji AK, Guha SK, Banerjee D, Chaudhuri D. Recurrence of kala-azar after PKDL: role of co-factors. Tropical medicine & international health: TM & IH. 1998;3(1):76–8.
- Gasim S, Elhassan AM, Khalil EA, Ismail A, Kadaru AM, Kharazmi A, Theander TG. High levels of plasma IL-10 and expression of IL-10 by keratinocytes during visceral leishmaniasis predict subsequent development of post-kala-azar dermal leishmaniasis. Clinical and experimental immunology. 1998;111(1):64–9.

## Supporting information

S1 Text **Table A.** Comparison of VL and PKDL: clinical, parasitological and immunological characteristics. **Table B.** Overview of studies performed in Sudan in VL patients that reported on subsequent PKDL rates. **Table C.** Incidence of PKDL by various VL treatment regimens, age and gender, in Bihar, India [adapted from ref [33]]. **Table D.** Incidence of PKDL by treatment regimens in Bangladesh [adapted from [34]].
(DOCX)
