# Supplementary material for: Risk factors for post-kala-azar dermal leishmaniasis (PKDL): Challenges in understanding pathophysiology
Source: PLoS Negl Trop Dis. 2026 Feb 23;20(2):e0013952. doi: 10.1371/journal.pntd.0013952 (PMC12928490; doi:10.1371/journal.pntd.0013952)
Supplement: S1 Text — Table A. Comparison of VL and PKDL: clinical, parasitological and immunological characteristics. Table B. Overview of studies performed in Sudan in VL patients that reported on subsequent PKDL rates. Table C. Incidence of PKDL by various VL treatment regimens, age and gender, in Bihar, India [adapted from ref [33]]. Table D. Incidence of PKDL by treatment regimens in Bangladesh [adapted from [34]]. (DOCX) [file pntd.0013952.s001.docx]

Supporting information S1 text

|  | VL | PKDL | Cure |
| --- | --- | --- | --- |
| Ill | yes | no | no |
| Skin lesions | no | yes | no |
| Infection | systemic | skin | no |
| Parasites | numerous | few | no |
| Hepatosplenomegaly | yes | no (usually) | no |
| Weight loss | yes | no | no |
| Immune response (predominant) | Th2 | Th2/ Th1 | Th1 |
| Leishmanin skin test (LST) | negative | positive or negative | positive |
| Positive serological test | yes | yes | yes |

Table A Comparison of VL and PKDL: clinical, parasitological and immunological characteristics.

| Recent data | Study | VL cure rate | PKDL | | Reference |
| --- | --- | --- | --- | --- | --- |
|  |  |  | rate | follow-up |  |
| SSG 20 mg/kg x 30 days | RCT | 188/200 (94%) | 13% | 6 m | (1) |
| SSG 20 mg/kg + paromomycin 15 mg/kg x 17 days | RCT | 328/359 (91%) | 6% | 6 m |  |
| Paromomycin 15 mg/kg x 21 days | RCT | 167/198 (84%) | 9% | 6 m |  |
|  |  |  |  |  |  |
| Paromomycin 15 mg/kg x 28 days | RCT | 17/21 (81%) | 19% | 6 m | (2) |
| Paromomycin 20 mg/kg x 21 days | RCT | 16/21 (81%) | 4% | 6 m |  |
|  |  |  |  |  |  |
| Paromomycin 20 mg/kg x 14 days + miltefosine x 14 days | RCT | 155/170 (91%) | 3% | 6 m | (3) |
| SSG 20 mg/kg x 17 days + paromomycin 15 mg/kg x 17 days | RCT | 156/170 (92%) | 14% | 6 m |  |
|  |  |  |  |  |  |
| AmBisome 10 mg/kg single dose + SSG 20 mg/kg x 10 days | RCT | 47/51 (87%) | 4% | 6 m | (4) |
| AmBisome 10 mg/kg single dose + miltefosine 2.5 mg/kg x 10 days | RCT | 40/45 (77%) | 2% | 6 m |  |
| Miltefosine 2.5 mg/kg x 10 days | RCT | 38/51 (72%) | 10% | 6 m |  |

Table B. Overview of studies performed in Sudan in VL patients that reported on subsequent PKDL rates

SSG sodium stibogluconate

RCT randomized controlled trial

6 m 6 months

|  | PKDL |  |
| --- | --- | --- |
| Characteristic | Hazard ratio (95%) CI | P value |
| VL treatment |  |  |
| Single dose AmBisome | Referent |  |
| AmBisome - Miltefosine | 1.27 (0.72, 2.22) | 0.4115 |
| Miltesosine - Paromomycin | 2.10 (1.37, 3.22) | 0.006 |
| Age |  |  |
| ≤ 12 years | 1.85 (1.25, 2.72) | 0.002 |
| > 12 years | Referent |  |
| Female | 1.91 (1.29, 2.81) | 0.0011 |
| Male | Referent |  |

Table C. Incidence of PKDL by various VL treatment regimens, age and gender, in Bihar, India. [adapted from ref (5)]

| Treatment regimen | No of participants | PKDL cases | Incidence rate in 100-person-years for 4 years (95% CI) |
| --- | --- | --- | --- |
| SSG | 168 | 5 | 3.0 (1.3, 7.3) ^a^ |
| MDAMB | 113 | 9 | 8.2 (4.3, 15.7) ^b^ |
| MF | 150 | 14 | 9.7 (5.7, 16.4) ^a,c^ |
| AMB + PMIM | 112 | 12 | 11.3 (6.4,19.9) ^a,d^ |
| SDAMB | 126 | 20 | 16.9 (10.9, 26.2) ^a^ |
| AMB + MF | 105 | 17 | 17.1 (10.6, 27.5) ^a^ |
| PMIM | 105 | 20 | 20.1 (13.0, 31.2) ^a,b,c^ |
| PMIM + MF | 105 | 24 | 25.3 (16.9, 37.7) ^a,b,c,d^ |
|  |  |  |  |
| Total | 984 | 121 | 14.0 (8.6, 22.7) |

Table D. Incidence of PKDL by treatment regimens in Bangladesh [adapted from (6)]

^a^ indicates SSG versus others with p< 0.05

^b^ indicates MDAMB versus others with p<0.05

^c^ indicates MF versus others with p<0.05

^d^ indicates AMB + PM versus others with p<0.05

SSG sodium stibogluconate

MDAMB multidose AmBisome

MF miltefosine

AMB AmBisome

SDAMB Single dose AmBisome

PMIM paromomycin Intramuscular

References

1. Musa A, Khalil E, Hailu A, Olobo J, Balasegaram M, Omollo R, et al. Sodium stibogluconate (SSG) & paromomycin combination compared to SSG for visceral leishmaniasis in East Africa: a randomised controlled trial. PLoS neglected tropical diseases. 2012;6(6):e1674.

2. Musa AM, Younis B, Fadlalla A, Royce C, Balasegaram M, Wasunna M, et al. Paromomycin for the treatment of visceral leishmaniasis in Sudan: a randomized, open-label, dose-finding study. PLoS neglected tropical diseases. 2010;4(10):e855.

3. Musa AM, Mbui J, Mohammed R, Olobo J, Ritmeijer K, Alcoba G, et al. Paromomycin and Miltefosine Combination as an Alternative to Treat Patients With Visceral Leishmaniasis in Eastern Africa: A Randomized, Controlled, Multicountry Trial. Clinical infectious diseases : an official publication of the Infectious Diseases Society of America. 2023;76(3):e1177-e85.

4. Wasunna M, Njenga S, Balasegaram M, Alexander N, Omollo R, Edwards T, et al. Efficacy and Safety of AmBisome in Combination with Sodium Stibogluconate or Miltefosine and Miltefosine Monotherapy for African Visceral Leishmaniasis: Phase II Randomized Trial. PLoS Negl Trop Dis. 2016;10(9):e0004880.

5. Goyal V, Das VNR, Singh SN, Singh RS, Pandey K, Verma N, et al. Long-term incidence of relapse and post-kala-azar dermal leishmaniasis after three different visceral leishmaniasis treatment regimens in Bihar, India. PLoS neglected tropical diseases. 2020;14(7):e0008429.

6. Mondal D, Kumar A, Sharma A, Ahmed MM, Hasnain MG, Alim A, et al. Relationship between treatment regimens for visceral leishmaniasis and development of post-kala-azar dermal leishmaniasis and visceral leishmaniasis relapse: A cohort study from Bangladesh. PLoS neglected tropical diseases. 2019;13(8):e0007653.
